# Obesity and depressive symptoms in mid-life: a population-based cohort study

**DOI:** 10.1186/s12888-018-1877-6

**Published:** 2018-09-17

**Authors:** Anwar Mulugeta, Ang Zhou, Christine Power, Elina Hyppönen

**Affiliations:** 1 Australian Centre for Precision Health, University of South Australia Cancer Research Institute, GPO Box 2471, Adelaide, SA 5001 Australia; 2 Department of Pharmacology, School of Medicine, College of Health Science, Addis Ababa University, Addis Ababa, Ethiopia; 3 Population, Policy and Practice, UCL Great Ormond Street Institute of Child Health, London, UK

**Keywords:** General obesity, Central obesity, Depressive symptoms, Middle-age

## Abstract

**Background:**

Obesity and depression are both highly prevalent public health disorders and evidence on their relationship is inconsistent. This study examined whether depressive symptoms are associated with current obesity, and further, whether obesity in turn is associated with an increased odds of depressive symptoms five years later after accounting for potential lifestyle confounders and depressive symptoms at baseline.

**Methods:**

Data were obtained from the 1958 British birth cohort (*N* = 9217 for cross-sectional and 7340 for prospective analysis). Clinical Interview Schedule-Revised and Mental Health Inventory-5 were used for screening depressive symptoms at ages 45 and 50 years, respectively. General and central obesity were defined using measurements of body mass index (BMI) and waist circumference (WC) at 45 years, respectively.

**Results:**

There was a cross-sectional association between depressive symptoms and obesity: participants with ≥2 depressive symptoms had 31% (95%CI 11% to 55%) higher odds of general and 26% higher odds of central obesity (95%CI 8% to 47%). In prospective analyses, both general and central obesity were associated with higher odds of depressive symptoms five years later among women but not in men (P_interaction_ < 0.01). After adjustment for depressive symptoms at baseline, sociodemographic and lifestyle factors, women with general obesity had 38% (95% CI 7% to 77%) and women with central obesity 34% (95%CI 9% to 65%) higher odds of depression compared to others.

**Conclusions:**

Depressive symptoms are associated with concurrent obesity and related lifestyle factors among women and men in mid-life. Our study suggests that obesity in turn affects long-term risk of depressive symptoms in women but not in men, independently of concurrent associations, providing an important target group for the implementation of preventative strategies.

## Background

Obesity and depression are both highly prevalent public health disorders that affect many age groups and communities [1, 2]. Globally, more than 600 million people are living with obesity while depression affects over 350 million people [1, 3]. Obesity and depression are interrelated; both are known risk factors for cardiovascular disorders and diabetes [4–6], and relate to negative health and lifestyles factors such as disturbed sleep patterns, sedentary behaviours and dysregulation in appetite and food intake [7]. These interrelations are clearly complex as use of antidepressant medication often leads to weight gain, as suggested from a review of ten clinical studies that explored the effect of antidepressants on body weight [8]. Furthermore, as evidenced by clinical trials conducted on women [9], dietary restriction targeting weight reduction in obesity may in turn exacerbate depression.

Previous observational studies on the association between obesity and depressive symptoms have provided mixed findings [10–17], with some suggesting a positive association [10], while others have reported an inverse association (obesity associated with lower risk of depression) [12], no association [13], or a u-shaped association (higher risk of depressive symptoms among underweight and obese individuals) [14]. These inconsistencies can arise from differences in population characteristics (for example, ethnicity and age) [12, 15, 18], residual confounding [19, 20], or potentially, differences in measures used to define obesity [21]. For example, a study of 3666 individuals from different ethnic groups in USA suggested an association among White but not Black or Mexican Americans [18]. A meta-analysis of 13 cross-sectional obesity-depression association studies (median *n* = 1215), indicated differences in point estimates and statistical precision based on the number of confounders included in the adjustment [20]. Furthermore, most previous studies consider “general” obesity, as measured by body mass index (BMI) [22, 23], while notably less attention has been paid to potential differences in risk based on body fat distribution. While BMI is widely used, it has well-known limitations and may reflect differences in muscle rather than adipose tissue [21]. Some studies have associated central obesity, as measured by waist circumference (WC) or waist-hip ratio (WHR), with depression risk [24]. Adipose tissue is an active endocrine organ associated with increased inflammatory response which relates to depression [25]. White adipose tissue which accumulates typically around the waist is a source of a range of inflammatory markers, which can pass the brain-blood barrier and thereby affect depression risk [26]. Another possibility is that dysregulation of the hypothalamus-pituitary-adrenal (HPA) axis could increase the accumulation of fat around the abdomen as well as leading to alterations in mood [27], possibly indicating a greater importance of central rather than general obesity with respect to depression risk.

This study used data from a large-scale population-based cohort to establish whether depressive symptoms in mid-adulthood are associated with concurrent general or central obesity and related lifestyle factors, and further, whether obesity in turn predicts the longer term risk of depressive symptoms after accounting for potential sociodemographic and lifestyle confounders and the presence of depressive symptoms at baseline.

## Methods

### Study population

This study used data from 1958 British birth cohort, which included all born in one week of 1958 in England, Scotland and Wales (*N* = 17,638) with immigrants born the same week included up to age 16 years (*N* = 920) [28]. The cohort has been followed up until adulthood, and this study primarily used data from the biomedical survey conducted at age 45 years (target sample 11,971, data collected for 9377) and from the 50 year follow-up [29].

**Fig. 1:**
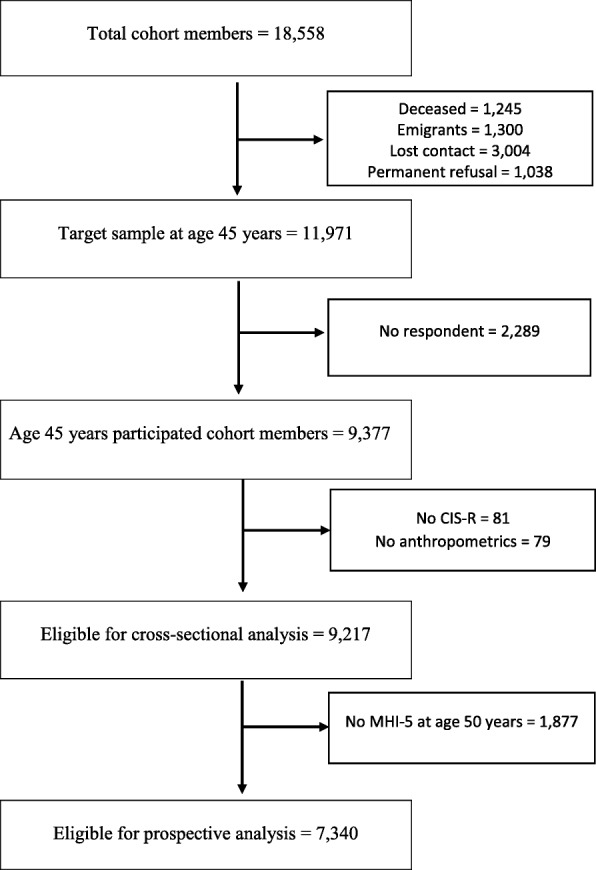
Flow diagram of the 1958 British Birth cohort for cross-sectional and prospective study

Participants with complete information on depressive symptoms, general and central obesity at age 45 years were included for cross-sectional analysis (*N* = 9217). Individuals from the cross-sectional analysis who also had information on depressive symptoms at 50 years (*N* = 7340) were included in the prospective analysis. The flow diagram for the selection of participants is shown in Fig. 1.

### Depressive symptoms

Depressive symptoms at 45 years were assessed using the Clinical Interview Schedule-Revised (CIS-R) [28]. CIS-R was developed for use by lay interviewers as a screening instrument for 14 common mental disorders including depression [30]. CIS-R has been reported to have an acceptable agreement with the Schedule for Clinical Assessment in Neuropsychiatry (SCAN), a clinical diagnostic tool for depression [31]. In the 1958 cohort, the CIS-R was administered by trained nurses; depressive symptoms were assessed using four questions that reflect symptom occurrence over the past week. These include “In the past week have you been able to enjoy or take an interest in things as much as usual?”, “In the past week on how many days have you felt sad, miserable or depressed/unable to enjoy or taken an interest in things?”, “Did you feel depressed for more than three hours in any day of the week?”, and “In the last week during depressive periods, did you feel happier when nice things happened or when you spent time with your friends?”. Participants presenting with two or more depressive symptoms were considered as case in our analyses [32, 33].

Mental Health Inventory − 5 (MHI-5), a short version of MHI-38, was used to assess depressive symptoms as part of the postal survey conducted at age 50 years [29] MHI-5 shows an acceptable validity in screening for depression compared to clinical depression diagnostic tools and has an equivalent level of performance in screening depression compared to other screening tools [34, 35]. MHI-5 has five self-administered questions which assess the amount of time in the past four weeks when the participants felt ^1)^ happy, ^2)^ calm and cheerful ^3)^ nervous, ^4)^ felt down and low or ^5)^ felt like nothing could cheer them up. Each question was rated using six Likert scale options (ranging from none to all of the time) to give a total score range of 5 to 30 [35]. Where needed, scores were converted to have lower values indicating a greater prevalence of symptoms and then scaled to 100. Scores ≤52 were used to indicate clinically significant depressive symptoms [34].

### Anthropometric measures

At age 45 years, height, weight and waist circumference were measured by trained nurses [36]. Weight (in light clothing) was measured with Tanita solar scales, and height (without shoes) with a stadiometer. Using a body tension tape, waist circumference was measured (over light clothing) between the lower ribs and iliac crest in the mid-axillary line [36]. BMI was calculated as weight (kg) over height squared (m^2^). According to WHO classification, BMI was categorized into four groups and coded as underweight (< 18.5), normal (≥18.5 and < 25), overweight (≥25 and < 30) and obesity (≥30) [37]. Central obesity was defined as waist WC ≥102 cm for males and ≥ 88 cm for females [38].

### Covariates

Sociodemographic and lifestyle factors were taken from age 45 years or from the nearest possible age sweep where the information was provided. Region of residence at age 45 years was coded as South, Middle and Northern England, London and Scotland; highest educational level attained by 33 years was grouped as <O level (less than Secondary education), O level, A level or higher (further education) [39]; socioeconomic position at birth (based on father’s occupation, or if missing, from age 7 years) and in adulthood (42 years) was categorised using the Registrar General Classification, and coded as I and II (professional/managerial), IIINM (skilled non-manual), IIIM (skilled manual) and IV and V (partially skilled and unskilled) [40, 41]. Information on smoking (currently non-smoker, ex-smoker or smoker), and physical activity (4–7 times/week, 2–3 times/week, 1 time/week and < 2–3 times/month), fruit consumption (< 1, 1–2, and ≥ 3 days/week) were collected at 42 years. Alcohol consumption (non-drinker; light drinker, < 7 units/week; moderate drinker, 7–14 units/weeks; heavy drinker, > 14 units/week), and sedentary behaviour based on time spent viewing a TV or PC (≥ 3 h and < 3 h/day) were from the 45 year survey.

### Statistical analysis

Logistic regression was used to evaluate both the cross-sectional and prospective associations. Cross-sectional analyses of depressive symptoms and obesity (general and central) included adjustments for sex, region, and social class at birth and in adulthood. Prospective analyses of obesity at 45 years and depressive symptoms at 50 years, included adjustments for sex and baseline depressive symptoms (model 1), with further adjustment for sociodemographic factors (region, social class at birth and in adulthood; model 2), and for both sociodemographic, and lifestyle factors (full model, further including alcohol, smoking, sedentary behaviour, physical activity, and fruit consumption; model 3). Models 1 to 3 were repeated for BMI and WC as continuous variables, using linear regression, to provide trend estimates and to test for curvature (using a quadratic term) of relationships with depression. BMI and WC were analysed separately and also mutually adjusted, to assess their independent effects. Interaction by sex was tested in cross-sectional and prospective analyses, and where found, results are presented separately for men and women.

Information on one or more social or lifestyle covariates was missing for 7% of cohort members. We used multiple imputation with chained equations (ice command in Stata) and present results from pooled analyses of 15 imputed datasets. There were no notable differences in the effect sizes obtained by analyses using multiple imputation compared to complete case analyses, although associations from models with multiple imputation were typically slightly more precise. All analyses were performed using Stata/ SE 14 software.

## Results

At age 45 years, around a quarter of participants were classified as having general obesity and 35% with central obesity. The prevalence of depressive symptoms was 8.4% at 45 years and 12.4% at 50 years. Sociodemographic and lifestyle factors associated with greater prevalence of general and central obesity were mostly also associated with higher prevalence of depressive symptoms (Table 1).

**Table 1:** Characteristics and distributions of adult obesity and depressive symptoms by sociodemographic and lifestyle factors

Covariates	N (%)	BMI mean (SD)	General obesity N (%)	Central obesity N (%)	Depression^b^ age 45y N (%)	Depression^c^ age 50y N (%)^d^
All	9217	27.4 (5.0)	2251 (24.4)	3208 (34.8)	776 (8.4)	911 (12.4)
Gender						
Male	4591 (49.8)	27.8 (4.3)	1162 (25.3)	1503 (32.7)	322 (7.0)	369 (10.5)
Female	4626 (50.2)	26.9 (5.6)	1089 (23.5)	1705 (36.9)	454 (9.8)	542 (14.2)
P		< 0.001	0.05	< 0.001	< 0.001	< 0.001
Social class age 42						
I & II	3715 (40.3)	27.2 (4.7)	813 (21.9)	1188 (32.0)	231 (6.2)	287 (9.4)
IIINM	1916 (20.8)	26.9 (5.0)	419 (21.9)	663 (34.6)	172 (9.0)	222 (14.0)
IIIM	1711 (18.6)	28.1 (4.9)	493 (28.8)	598 (35.0)	127 (7.4)	150 (11.6)
IV & V	1469 (15.9)	27.8 (5.5)	423 (28.8)	593 (40.4)	167 (11.4)	183 (16.3)
Others	406 (4.4)	27.7 (5.6)	103 (25.4)	166 (40.9)	79 (19.5)	69 (25.8)
P^a^		< 0.001	< 0.001	< 0.001	< 0.001	< 0.001
Alcohol (units/week)						
Non-drinker	623 (6.8)	28.2 (6.3)	193 (31.0)	253 (40.6)	108 (17.3)	114 (24.8)
Light < 7	4476 (48.6)	27.6 (5.3)	1196 (26.9)	1, 642 (36.7)	367 (8.2)	428 (11.9)
Moderate 7–14	2226 (24.2)	26.8 (4.3)	431 (19.4)	679 (30.5)	137 (6.1)	185 (10.2)
Heavy > 14	1854(20.0)	27.3 (4.6)	214 (21.0)	616 (33.2)	160 (8.6)	182 (12.6)
Missing	38 (0.4)	27.3 (4.6)	10 (26.3)	18 (47.4)	4 (10.5)	2 (11.1)
P^a^		< 0.001	< 0.001	< 0.001	0.002	0.008
Smoking						
Never	4256 (46.1)	27.4 (5.0)	1024 (24.1)	1440 (33.8)	307 (7.2)	386 (10.9)
Ex- smoker	2497 (27.1)	27.8 (5.0)	645 (25.8)	914 (36.6)	185 (7.4)	217 (8.7)
Current smoker	2161 (23.5)	27.0 (5.1)	520 (24.1)	752 (34.8)	249 (11.5)	282 (13.1)
Missing	303 (3.3)	27.0 (4.7)	62 (20.5)	102 (33.7)	35 (11.6)	26 (14.1)
P^a^		0.49	0.31	0.05	< 0.001	< 0.001
Physical activity						
< 2–3 times/month^e^	3026 (32.8)	28.0 (5.5)	878 (29.0)	1229 (40.6)	317 (10.5)	352 (14.9)
1 time/week	1670 (18.1)	27.4 (4.8)	415 (24.9)	565 (33.8)	104 (6.2)	144 (10.4)
2–3 times/week	1932 (21.0)	27.1 (4.5)	413 (21.4)	612 (31.7)	138 (7.1)	161 (10.3)
4–7 times/week	2295 (24.9)	26.9 (4.9)	484 (21.1)	703 (30.6)	181 (7.9)	227 (12.2)
Missing	294 (3.2)	26.9 (4.3)	61 (20.8)	99 (33.7)	36 (12.2)	27 (15.3)
P^a^		< 0.001	< 0.001	< 0.001	< 0.001	0.003
Sedentary behaviour						
< 3 h/day	5806 (63.0)	26.9 (4.7)	1195 (20.6)	1751 (30.2)	429 (7.4)	517 (11.0)
≥ 3 h/day	2995 (32.5)	28.4 (5.4)	945 (31.6)	1296 (43.3)	293 (9.8)	348 (14.9)
Missing	416 (4.5)	27.7 (5.4)	111 (26.7)	161 (38.7)	54 (13.0)	46 (16.7)
P^a^		< 0.001	< 0.001	< 0.001	< 0.001	< 0.001
Fruit consumption						
< 1 day/week	1676 (18.2)	27.5 (5.0)	433 (25.8)	618 (36.9)	195 (11.6)	220 (17.5)
1–2 days/week	1367 (14.8)	27.5 (5.1)	349 (25.5)	506 (37.0)	113 (8.3)	161 (15.1)
≥ 3 days/week	5879 (63.8)	27.4 (5.0)	1407 (23.9)	1984 (33.8)	432 (7.4)	503 (10.4)
Missing	295 (3.2)	27.0 (4.3)	62 (21.0)	100 (33.9)	36 (12.2)	27 (15.3)
P^a^		0.74	0.13	0.001	< 0.001	< 0.001

^a^*P*-value from logistic regression adjusted for sex

^b^Depressive symptoms measured using CIS-R

^c^Depressive symptoms measured using MHI-5. ^d^ Cohort members who had missing data on depressive symptoms at age 50 (n = 1, 877) have been excluded. ^e^ include individuals who responded no for “Do you do any regular exercise?”

Table 2 shows the cross-sectional associations of depressive symptoms with obesity and lifestyle factors at 45 years. The odds of obesity were higher for those reporting ≥2 symptoms compared to individuals with no depressive symptoms (OR 1.31, 95%CI 1.11 to 1.55 and OR 1.26, 95%CI 1.08 to 1.47, for general and central obesity, respectively). The cross-sectional associations between depressive symptoms and general or central obesity did not vary by sex (P _sex-interaction_ > 0.33). Number of depressive symptoms was also related to sedentary behaviour, smoking and low fruit consumption (*P* < 0.001 for all comparisons after adjustment for sex, region, social class at birth and in adulthood). The association of depressive symptoms with cigarette smoking and fruit consumption varied by sex (P_sex-interaction_ < 0.02 for both comparisons), and both associations were observed in women but not men. Compared to women without symptoms, those with symptoms at baseline had an OR 1.82 (95% CI 1.38 to 2.19; men OR 1.05, 95% CI 0.78 to 1.40) for smoking and OR 0.51 (95% CI 0.40 to 0.67; men OR 0.76, 95% CI 0.57 to 1.00) for fruit consumption.

**Table 2:** Cross-sectional association of depressive symptoms with obesity and lifestyle factors at age 45 years

		Obesity, OR (95% CI)^a^		Lifestyle factors^b^, OR (95% CI)^a^			
	N^c^ (%)	General obesity	Central obesity	Sedentary behaviour	High alcohol consumption	Current smoking	Fruit consumption
Depressive symptoms							
None	7683 (83.4)	Reference	Reference	Reference	Reference	Reference	Reference
1	758 (8.2)	1.07 (0.90, 1.27)	1.02 (0.87, 1.19)	1.36 (1.16, 1.59)	1.15 (0.95, 1.40)	1.47 (1.23, 1.74)	0.82 (0.68, 1.00)
≥ 2	776 (8.4)	1.31 (1.11, 1.55)	1.26 (1.08, 1.47)	1.30 (1.10, 1.53)	1.19 (0.99, 1.44)	1.53 (1.30, 1.82)	0.52 (0.53, 0.76)
P_unadjusted_		0.001	< 0.001	< 0.001	0.89	< 0.001	< 0.001
P_adjusted_^a^		0.005	0.01	< 0.001	0.08	< 0.001	< 0.001
P_sex-interaction_^d^		0.33	0.58	0.52	0.11	< 0.001	0.02

^a^*P*-value adjusted and OR (95% CI): adjustment made for sex, region, social class at birth and social class at age 42 years

^b^Sedentary behaviour, spent 3 or more hrs/day viewing a TV or PC; high alcohol consumption, > 15 units/week alcohol consumption; current smoking, smoking during age 42 sweep; fruit consumption, < 1 day/week fruit consumption vs > 1 day/week fruit consumption

^c^All the analysis done in 9217 sample population of the age 45

^d^Interaction between sex and depressive symptoms on obesity and on lifestyle factors after adjustment for sex, region, social class at birth and social class at age 42 years

Both general and central obesity at 45 years were associated with higher odds of depressive symptoms at age 50 years in women but not in men (Table 3, P_sex-interaction_ < 0.01 for all comparisons). In women, the observed associations between BMI and WC with depressive symptoms were somewhat attenuated by adjustment for sociodemographic and lifestyle factors, but for both obesity indicators the associations remained even after accounting for baseline symptoms, sociodemographic and lifestyle factors (BMI: OR per 10 kg/m^2^ 1.36, 95%CI 1.15 to 1.62; WC: OR per 5 cm 1.07, 95%CI 1.03 to 1.11). In men the fully adjusted OR for BMI was 0.83 (95%CI 0.64 to 1.10, P_trend_ *= 0.20)* and for WC 0.99 (95%CI 0.94 to 1.04, P_trend_ = 0.67). In a model including all covariates and mutually adjusted for BMI and WC, the association in women for BMI was abolished (OR 0.96, 95%CI 0.77 to 1.18) while the estimate for WC remained significant (OR 1.08, 95%CI 1.02 to 1.15). Again, the corresponding mutually adjusted model among men did not suggest an association either for WC or BMI (*P* > 0.16 for all comparisons).

**Table 3:** Prospective association between general and central obesity at age 45 years and depressive symptoms at age 50 years (*n* = 7340) among men and women

	Women			Men		
	Model-1 OR (95% CI)	Model-2 OR (95% CI)	Model-3 OR (95% CI)	Model-1 OR (95% CI)	Model-2 OR (95% CI)	Model-3 OR (95% CI)
Body Mass Index						
Underweight	0.72 (0.21, 2.49)	0.72 (0.20, 2.50)	0.54 (0.15, 1.99)	2.47 (0.45, 13.05)	2.30 (0.41, 12.80)	1.91 (0.33, 11.21)
Normal	Reference	Reference	Reference	Reference	Reference	Reference
Overweight	1.11 (0.87, 1.40)	1.08 (0.85, 1.38)	1.10 (0.86, 1.40)	0.87 (0.64, 1.13)	0.87 (0.65, 1.15)	0.86 (0.65, 1.15)
Obesity^a^	1.56 (1.22, 1.98)	1.46 (1.14, 1.86)	1.38 (1.07, 1.77)	0.87 (0.63, 1.21)	0.87 (0.63, 1.21)	0.85 (0.61, 1.18)
Per 10 kg/m^2^	1.48 (1.26, 1.74)	1.41 (1.20, 1.67)	1.36 (1.15, 1.62)	0.87 (0.66,1.14)	0.86 (0.65,1.12)	0.83 (0.64,1.10)
P_trend_	< 0.001	< 0.001	< 0.001	0.31	0.26	0.20
P_curvature_	0.25	0.19	0.32	0.05	0.11	0.14
Waist circumference						
Normal	Reference	Reference	Reference	Reference	Reference	Reference
Central obesity^b^	1.48 (1.21, 1.81)	1.41 (1.15, 1.73)	1.34 (1.09, 1.65)	0.97 (0.75, 1.23)	0.94 (0.73, 1.21)	0.90 (0.70, 1.17)
Per 5 cm	1.10 (1.06, 1.14)	1.09 (1.05, 1.13)	1.07 (1.03, 1.11)	1.00 (0.95, 1.05)	1.00 (0.95, 1.05)	0.99 (0.94, 1.04)
P_trend_	< 0.001	< 0.001	< 0.001	0.99	0.88	0.67

P_sex-interaction_ is the interaction between sex and obesity variables at age 45 years on depression at age 50 years (For all obesity variables, P_sex-interaction_ < 0.01)

Model-1, adjusted for depressive symptoms at age 45 years; Model-2, adjusted for depressive symptoms at age 45 years, and region, social class at birth and social class at adulthood; Model-3, adjusted for depressive symptoms at age 45 years, region, social class at birth, social class at adulthood, fruit consumption, physical activity, sedentary behaviour, smoking, alcohol)

^a^BMI ≥ 30 kg/m^2^

^b^WC ≥ 102 cm for men and WC ≥ 88 cm for women

To further understand the association between obesity and depressive symptoms, we explored the prospective associations between lifestyle factors and depressive symptoms. Alcohol consumption, smoking, sedentary behaviour and fruit consumption were all associated with depressive symptoms at 50 years after adjustment for depressive symptoms at 45 years, sex, sociodemographic and other lifestyle factors (Table 4). The presence of depressive symptoms at age 45 years was associated with an over four-fold greater odds of depressive symptoms at 50 years, attenuating only slightly after adjustment for obesity and lifestyle factors (OR reduced from 4.43 (95%CI 3.61 to 5.43) to 4.11 (95%CI 3.34 to 5.06)), suggesting further unmeasured influences on the tracking of depressive symptoms.

**Table 4:** Lifestyle factors and the risk of depressive symptoms at age 50 years

	Adjusted for sex, depressive symptoms at 45y, & other factors^a^	Full adjustment^b^	
	OR(95%CI)	OR(95%CI)	P_sex-interaction_
Alcohol			0.40
Non-drinker	1.98 (1.52, 2.58)	1.88 (1.44, 2.46)	
Light < 7 units/week	Reference	Reference	
Moderate 7–14 units/week	0.98 (0.80, 1.19)	1.00 (0.82, 1.22)	
Heavy > 14 units/week	1.23 (1.00, 1.51)	1.17 (0.95, 1.45)	
P	< 0.001	< 0.001	
Smoking			0.05
Never	Reference	Reference	
Ex-Smoker	0.96 (0.80, 1.16)	0.93 (0.77, 1.13)	
Current smoker	1.51 (1.25, 1.81)	1.33 (1.09, 1.61)	
P	< 0.001	0.003	
Physical activity			0.51
2–3 times/month	Reference	Reference	
1 time/week	0.79 (0.63, 0.98)	0.88 (0.71, 1.10)	
2–3 times/week	0.76 (0.61, 0.94)	0.88 (0.70, 1.09)	
4–7 times/week	0.86 (0.71, 1.05)	0.98 (0.81, 1.20)	
P	0.04	0.52	
Sedentary behaviour			0.09
< 3 h/day	Reference	Reference	
≥ 3 h/day	1.36 (1.16, 1.59)	1.20 (1.02, 1.41)	
P	< 0.001	0.05	
Fruit consumption			0.02
< 1 day/week	Reference	Reference	
1–2 days/week	0.93 (0.73, 1.18)	0.98 (0.76, 1.25)	
≥ 3 days/week	0.60 (0.49, 0.73)	0.67 (0.55, 0.82)	
P	< 0.001	< 0.001	
Depressive symptoms at age 45y			0.36
No	Reference	Reference	
Yes	4.43 (3.61, 5.43)	4.11 (3.34, 5.0)	
P	< 0.001	< 0.001	

P-value based on Likelihood ratio test

^a^Sociodemographic factors included region, social class at birth and social class at age 42 years

^b^Full adjustment involved sex, depressive symptoms at age 45 years, sociodemographic factors, general and central obesity, alcohol, smoking, physical activity, sedentary behaviour, and fruit consumptions

## Discussion

Tackling the current obesity epidemic both in terms of addressing its causes and consequences remains a key public health priority. Mental wellbeing has broad influences on health and as shown in our large-scale study, the presence of depressive symptoms is strongly associated both with obesity and a broad range of related lifestyle factors. However, as shown in our analyses, even after controlling for these concurrent associations, obesity several years earlier appears to affect subsequent odds of depressive symptoms among women but not in men, providing an important target group for implementation of preventative strategies. Furthermore, this study provided some support for the hypothesis that central rather than general adiposity may have a key role in contributing to the association between obesity and the risk of developing depressive symptoms, highlighting the need of further studies to establish related mechanisms and pathways.

Our work builds on an earlier analysis in the 1958 cohort, which suggested that when looking at categories of BMI from childhood to adulthood (age 7 onwards) obesity was associated with the subsequent odds of depressive symptoms in women but not in men [15]. Inclusion here of central obesity is important because BMI has known weaknesses, especially for men, due to the strong correlation with muscle mass [21]. The result from our previous work [15] is consistent with the current study, and both the analysis using BMI (or general obesity) and WC (or central obesity) suggest that obesity is affecting the likelihood of developing depressive symptoms in women only.

Our findings for higher odds of depressive symptoms by general and central obesity are consistent with several other previous studies [20, 22]. For example, a meta-analysis including eight cross-sectional studies reported the odds of depression to be 35% higher in people with obesity compared to normal weight individuals [42]. A meta-analysis reporting prospective associations also included eight studies, six of them assessed to be of poor quality by the authors, suggesting an overall 55% higher odds of depression for those with obesity compared to normal weight, with no evidence for sub-group differences by sex [22]. Another meta-analysis including 15 studies showed 38% higher odds of depression in individuals with central obesity compared to those without, with higher estimates among women than men [20]. Importantly, unlike these previous studies [20, 22], our study included an adjustment for depressive symptoms at baseline. Furthermore, the weaker effect estimate in our study compared to previous meta-analyses may be due to better control for sociodemographic or other confounding factors as most previous studies included in the two meta-analyses [20, 22, 42] were either unadjusted or adjusted for limited set of confounders.

We observed a dose-dependent increase in the odds of depressive symptoms by categories of BMI, which is consistent with most previous studies [10, 13, 43]. However, in some studies, the association between general obesity and depressive symptoms has favoured the “Fat and Jolly” hypothesis, suggesting that obesity may protect against depression [44] while others show a higher odds both for the very thin and obese individuals compared to those with normal weight [14, 16, 45]. It is possible that an increased odds of depression for those who are thin or underweight, can reflect sub-optimal health status [46]. These inconsistencies among studies could be further explained by contextual, and psychosocial influences contributing to the obesity – depression relationship. Individuals who experience stigma and discrimination due to their weight may have low self-esteem and high degree body image dissatisfaction that further aggravate psychological stress and lead to depressive symptoms [47]. Indeed, the possible stigma and discrimination due to excessive body weight is likely to have a greater impact on women than men which may explain the observed gender difference [47].

Mechanisms underlying the association between obesity and depression remains unclear, however, molecular and clinical studies have provided some evidence for the involvement of HPA axis, inflammatory pathways and insulin sensitivity [48], with dysregulation of HPA axis, elevation of certain inflammatory markers and insulin resistance being observed in both individuals with obesity and in individuals with depression [49–51]. Disturbance in any of these systems may affect the secretion or metabolism of neurotransmitters, such as serotonin, norepinephrine and dopamine, in the brain and consequently influence mood [48, 52]. Findings regarding inflammatory pathways and insulin sensitivity are particularly interesting: the observed elevated inflammatory markers, including leptin, adiponectin and IL-6 and TNF-α are mainly secreted from white adipose tissue found on the abdomen [26]; abdominal obesity, in particular, places people at higher risk for developing insulin resistance [53]. All these observations resonate with our findings of an independent association for WC and highlight the particular importance of central obesity on the risk of developing depressive symptoms.

Our study also suggests that the association between obesity and depressive symptoms is in part related to socioeconomic and lifestyle factors, with obesity associations attenuating by about 30% after allowing for such factors. This attenuation was expected considering that most studies show lower socioeconomic status and unhealthy lifestyle factors to be associated with obesity and depression [54, 55] although some factors may be on the causal pathway from obesity to depressive symptoms. For example, obesity may lead to less active lifestyles as well as inactivity increasing the risk of obesity [56]. Even so, given the links shown here between lifestyle factors and depressive symptoms, our study suggests that these behaviours may be additional targets for interventions to reduce obesity and depression.

### Limitations

It is important to emphasise the following limitations of this study. Firstly, our study used the presence of depressive symptoms as the outcome rather than defining it based on the diagnosis of depressive disorder. In an earlier meta-analysis, the association between obesity and depression was found to be stronger for depressive disorder than for depressive symptoms [22]. This may explain why the associations found here are weaker than other reported estimates. Different instruments were used to assess depressive symptoms at ages 45 and 50 years, although both have been shown to be valid, with similar performance compared to standard diagnostic tools and both have been used in community-based epidemiological studies for screening of depressive symptoms [57, 58]. Another limitation relates to representativeness, as the ethnic composition of the current UK population is notably more diverse compared to the participants included here, 98% of whom were European origin [28]. Hence, the results may not be generalisable to today’s more ethnically diverse population in the UK. A further limitation relates to inadequately measured or unmeasured confounding: although a broad range of lifestyle and sociodemographic factors were included, some measures (e.g. sedentary behaviour, physical activity) may have limitations, whilst in total the measures may not have captured all variation influencing the association between obesity and depressive symptoms. Finally, our study is limited in its ability to fully dissect independent effects of general and central adiposity. BMI and WC are highly correlated, and while our analyses suggest that WC may be the major influence, further studies avoiding problems with collinearity are needed to confirm this interpretation. Alternative approaches such as Mendelian randomisation may provide further insights into the independent causal roles of central and general obesity on depression risk.

## Conclusion

Depressive symptoms are associated with obesity and related lifestyle factors in mid-life. Our study suggests that obesity affects subsequent risk of developing depressive symptoms among women independently of these concurrent associations, providing an important target group for implementation of preventative strategies. In the management of depressive symptoms, one of the alternative approaches could be targeting and managing obesity and unfavourable lifestyle factors.

## Abbreviations

BMI: Body Mass Index
CIS-R: Clinical Interview Schedule – Revised
HPA: Hypothalamus-Pituitary-Adrenal Axis
IL-6: InterLeukin-6
MHI-5: Mental Health Inventory-5
MHI-8: Mental Health Inventory-8
OR: Odds Ratio
PC: Personal Computer
SCAN: Schedule for Clinical Assessment in Neuropsychiatry
TNF-α: Tissue Necrotizing Factor—α
TV: Television
UK: United Kingdome
WC: Waist Circumference
WHO: World Health Organisation
WHR: Waist – Hip Ratio
